# Synchrony Degree of Dietary Energy and Nitrogen Release Influences Microbial Community, Fermentation, and Protein Synthesis in a Rumen Simulation System

**DOI:** 10.3390/microorganisms8020231

**Published:** 2020-02-09

**Authors:** Jun Zhang, Nan Zheng, Weijun Shen, Shengguo Zhao, Jiaqi Wang

**Affiliations:** 1 State Key Laboratory of Animal Nutrition, Institute of Animal Sciences, Chinese Academy of Agricultural Sciences, Beijing 100193, China; June_zh16@cau.edu.cn (J.Z.);; 2 College of Animal Science and Technology, Hunan Agricultural University, Changsha 410128, China

**Keywords:** synchrony, microbial protein synthesis, rumen, bacterial community, energy, nitrogen

## Abstract

Synchrony of energy and nitrogen release in rumen has been proposed to maximize ruminal microbial fermentation. However, the information regarding bacterial community composition and its metabolism under a higher or lower degree of synchronization is limited. In our study, a 0 to 6 h post-feeding infusion (first half infusion, FHI), 6 to 12 h post-feeding infusion (second half infusion, SHI), and 0 to 12 h post-feeding infusion (continuous infusion, CI) of maltodextrin were used to simulate varying degrees of synchronization of energy and nitrogen release in a rumen simulation system. In addition, the bacterial community, metabolite, enzyme activity, and microbial protein synthesis (MPS) were evaluated. Compared with the FHI and CI, the relative abundance of *Fibrobacter*, *Ruminobacter*, BF311, and CF231 decreased in the SHI, but that of *Klebsiella* and *Succinivibrio* increased in the SHI. The NH_3_-N and branched-chain volatile fatty acids were significantly higher, but propionate content and activities of glutamate dehydrogenase (GDH) and alanine dehydrogenase were significantly lower in the SHI than those in the FHI and CI. The SHI had lower MPS and less efficiency of MPS than the FHI and CI, which indicated that the SHI had a lower degree of synchronization. Correlation analysis showed that MPS was positively related to GDH activity and relative abundance of *Fibrobacter* but negatively related to NH_3_-N and relative abundance of *Klebsiella*. Therefore, a higher degree of synchronization of energy and nitrogen release increased MPS partly via influencing the bacterial community, metabolism, and enzyme activities of ammonia assimilation in the in vitro fermenters.

## 1. Introduction

Milk, especially milk protein, is an important nutrient source for most people. Milk protein has different types of biological activities, e.g., essential amino acids, growth factors, hormones, enzymes, antibodies, and immune stimulants [1]. An increasing supply of metabolizable protein can increase milk protein yield, and microbial crude protein (MCP) contributes about 40%–60% of metabolizable protein [2,3]. In addition, compared with feed protein, MCP is an excellent source for milk protein production in terms of amino acid contents [4]. Meanwhile, a better balance and utilization of ruminal degradable and undegradable protein can increase metabolizable protein and reduce nitrogen excretion in urine and feces [5]. Therefore, the improvement of ruminal microbial protein synthesis (MPS) not only benefits milk protein production, but also benefits nitrogen utilization efficiency, which avoids excessive emission of nitrogen to the environment [6,7].

Synchronizing the rate of supply of energy and nitrogen sources to rumen microorganisms has been proposed to maximize the capture of rumen degradable protein and to optimize microbial growth rate and efficiency [8,9,10]. Meanwhile, MPS and animal performance are critical standards to justify whether a treatment is more synchronous or not [10,11,12]. Synchrony has been studied for more than thirty years. However, the results of both in vitro and in vivo experiments have been inconsistent and some of the improvements are not seen in practice [11,13,14]. Berthiaume et al. (2010) and Henning et al. (1993) found that interaction between protein and energy release had no effect on microbial flow and growth efficiency, but the energy infusion group had more total and microbial nitrogen than the protein infusion group [15,16]. The degree of synchronization in the ruminal release of energy and nitrogen influences MPS when the rumen system is fed with certain diets such as those containing large amounts of fermentable carbohydrates [8,17,18]. Knowledge of synchrony of energy and protein release in rumen is helpful to guide dietary preparation to increase milk protein synthesis as well as nitrogen utilization efficiency, especially in the total mixed ration fed dairy cattle system.

Rumen microbes play key roles in the degradation of feedstuffs into volatile fatty acids (VFAs), amino acids, and peptides, which provide energy and nitrogen sources for both themselves and hosts [19]. In addition, microbes are very crucial in the maintenance, health, growth, welfare, and productivity of hosts [20,21]. To our knowledge, most previous studies did not pay much attention to the microbial community change in the more or less synchronous systems. Only conventional culture techniques [10] and real-time PCR [12] were used to examine the changes of several bacteria in more or less synchronous systems. No study inquired into uncultured bacteria, which is abundant in the rumen microbial community [22], in more or less synchronous systems. Hence, we still lack an integrated view of the composition and structure of the rumen microbial community within more or less synchronous systems. The next-generation sequencing method has been successfully used in microbial communities to explore ruminal bacterial ecology, providing the possibility to gain such an integrated view [23,24,25].

Thus, we hypothesized that varying degrees of synchronization can alter both the MPS and bacterial community in rumen. Therefore, by using infusions of maltodextrin for 0 to 6 h post-feeding, for 6 to 12 h post-feeding, or continuously through the day, this study aims to examine the impact of different degrees of synchronization of energy and nitrogen release on the bacterial community, metabolites, enzyme activity, and MPS in a rumen simulation system.

## 2. Materials and Methods

### 2.1. Experimental Design and Cultivation

**Figure 1:**
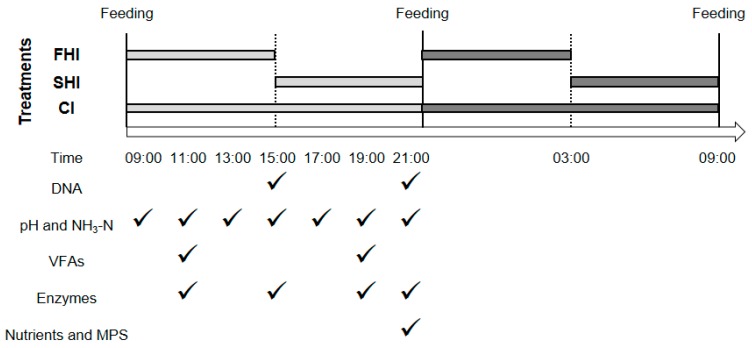
Schematic representation of treatment and sampling in this study. FHI treatment represents maltodextrin infusion from 09:00 to 15:00; SHI treatment represents maltodextrin infusion from 15:00 to 21:00; CI treatment represents maltodextrin infusion from 09:00 to 21:00. Light gray means infusion proceeded at daytime, and the dark gray means infusion proceeded at nighttime. Checkmark means sampling for corresponding items. FHI, first half infusion of maltodextrin; SHI, second half infusion of maltodextrin; CI, continuous infusion of maltodextrin; VFAs, volatile fatty acids; MPS, microbial protein synthesis.

The rumen simulation system with six fermenters described in Shen et al. (2012) was used in this study. In two replicated periods of 8 d (5 d for adaptation and 3 d for sampling) [26], two fermenters (nominal liquid volume 1000 ± 20 mL) were randomly assigned to each treatment in each period. The basic diet (Table 1) was ground through a 1-mm screen using a Wiley mill (standard model 4, Arthur H. Thomas Co., Philadelphia, PA, USA) and mixed thoroughly. The fermenters were manually fed with 40 g (DM basis) of diet per day, which was divided into two equal feedings at 09:00 and 21:00 h. Maltodextrin (degree of polymerization 6.7–12.5 with dextrose equivalent 8.0–15.0; Sinopharm Chemical Reagent Co., Ltd., Shanghai, China) was added to McDougall′s buffer [27]. The buffer was continuously infused into the fermenters with the dose of 10 g maltodextrin per day. The three treatments were defined as (1) first half infusion group (FHI): two separated 6 h infusions started at 09:00 and 21:00; (2) second half infusion group (SHI): two separated 6 h infusions started at 15:00 and 03:00; and (3) continuous infusion group (CI): 24-h infusion started at 09:00 to next day 09:00. In each 6 h infusion period of FHI and SHI, 480 mL buffer including 5 g of maltodextrin was infused into the fermenter. In each 24-h infusion period of CI, 1920 mL buffer including 10 g of maltodextrin was infused into the fermenter. The FHI, SHI, and CI, which had the same quantity of maltodextrin infused per day, were considered to mimic different release rates of energy matching the same release rate of nitrogen in the basal diet and then to form varied degrees of synchronous diets in fermenters. The schematic representation for treatments was shown in Figure 1.

Rumen fluid was collected at 2 h after morning feeding from three ruminal fistulated lactating Holstein dairy cows that were fed a mid-lactation diet (15.5% crude protein (CP), 30.2% neutral detergent fiber (NDF), assayed with a heat stable alpha-amylase and sodium sulfite and expressed inclusive of residual ash) consisting of 55% forage and 45% concentrate on a dry matter (DM) basis (Table 1), squeezed through four layers of cheesecloth into a sealed container, and transferred to the lab within 2 h. All procedures with cows were performed in accordance with the guidelines approved by the Animal Care and Use Committee for Livestock issued by the Institute of Animal Science, Chinese Academy of Agricultural Sciences (Beijing, China). A total of 500 mL of the strained ruminal fluid was added to each fermenter, which also contained 500 mL of McDougall’s buffer [27]. Before use, the McDougall’s buffer was continuously infused with CO_2_ [27], and 0.4 g/L urea was included in the buffer to mimic rumen nitrogen recycling [28,29]. Infusion of McDougall’s buffer and flow of filtered liquid were set to maintain solid and liquid dilution rates of approximately 4.0%/h and 8.0%/h, respectively, and the fermenter content was stirred continuously at 25 rpm [25,30]. Anaerobic conditions were established by flushing the headspace of the fermenters with N_2_ at a rate of 20 mL/min, and the temperature of the fermenters was maintained at 39 °C by circulating water [25,30].

### 2.2. Sampling

Samples of fermenter fluid (approximately 10 mL) were obtained directly from the built-in sampling pipe in the middle of each fermenter on days 6, 7, and 8 at 09:00 (before feeding), 11:00, 13:00, 15:00, 17:00, 19:00, and 21:00 h (before feeding). Fermenter fluid (1 mL) samples collected at 15:00 and 21:00 h were used for microbial total DNA extraction, which represents the middle and end stages of one fermentation cycle, respectively. The pH of the fermenter fluid was measured with a mobile pH meter (Starter 300; Ohaus, NJ, USA) immediately after collection. Fermenter fluid (1 mL) from all time points was added with 100 μL 6 M HCL to test NH_3_-N concentration. Fermenter fluid (1 mL) collected at 11:00 and 19:00 h (2 and 10 h after morning feeding, respectively) was added with 100 μL 25% metaphosphoric acid and then used to determine VFAs. The fermenter fluid (2 mL) from 11:00, 15:00, 19:00, and 21:00 h, which represents the early, middle, late, and end stages of one fermentation cycle, respectively, were collected and centrifuged at 12,000× *g* for 12 min at 4 °C. Then, the pellet was resuspended in PBS buffer and homogenized twice for 1 min at 30 MZ on Oscillating Mill MM 400 (Retsch, Hahn, Germany) with sterile zirconia beads (0.5 mm). The clear supernatant was used to determine the activity of ammonia assimilation enzymes including glutamine synthetase (GS), glutamate dehydrogenase (GDH), glutamate synthetase (GOGAT), and alanine dehydrogenase (ADH).

Effluent was collected every day from days 6 to 8 in a 1.0-L container immersed in an ice water bath and filtered through a nylon cloth (Guangda Hengyi Co., Beijing, China) with an inner size of 8 cm × 12 cm and a pore size of 40 μm, as described in previous studies [15,31]. The residues were used to determine DM, organic matter (OM), NDF and CP as described in other studies [32,33], and filtrate was used to detect MPS.

### 2.3. Microbial DNA Extraction and Quantitative PCR

Total DNA was extracted from 72 fermenter fluid samples using cetyltrimethylammonium bromide and the bead-beating method, as described previously [34]. Extracted DNA was assessed using agarose gel (1%) electrophoresis and quantified using a Qubit 2.0 Fluorometer (Thermo Scientific, Waltham, MA, USA). The quantitative PCR primer sets of total bacteria were 338-F (5′-ACTCCTACGGGAGGCAGCAG-3′) and 533-R (5′-TTACCGCGGCTGCTGGCAC-3′) as described by a previous study [35]. Quantitative PCR was performed using the ABI 7500 real time PCR system (Applied Biosystems, Carlsbad, CA, USA), similar to our previous study [34]. Each reaction contained 5 μL of Power SYBR Green PCR Master Mix (Takara Bio, Dalian, China), 1 μL of each primer (10 μM), 0.2 μL of Rox (Takara Bio), 25 ng of microbial DNA, and 2.3 μL of deionized water. Thermal cycling was performed at 95 °C for 30 s, followed by 40 cycles of 95 °C for 15 s, 55 °C for 34 s, and 72 °C for 1 min. In total, 72 samples were determined, and each sample was detected in triplicate. Standard curves were generated using the gradient diluted plasmids DNA, and the 16S rRNA gene copies of total bacteria were determined by relating the CT value to the standard curves.

### 2.4. Bacterial 16S rRNA Genes Amplification and Miseq Sequencing

The amplification of 16S rRNA genes from the 72 samples were done using the universal bacterial primers 515F (5′-GTGCCAGCMGCCGCGGTAA-3′) and 806R (5′-GGACTACHVGGGTWTCTAAT-3′) that are tagged with unique barcode sequences for each sample [36]. PCRs were carried out in 50 μL reactions with 0.5 μL of PrimeSTAR HS DNA Polymerase (Takara Bio, Dalian, China), 10 μL 5 × PrimeSTAR Buffer (plus Mg2^+^, Takara Bio, Dalian, China), 0.2 μM of the forward and reverse primers, 200 μM dNTP (Takara Bio, Dalian, China), and 100 ng microbial DNA. Amplification was performed as follows: initial denaturation at 95 °C for 1 min, 30 cycles of denaturation at 95 °C for 30 s, annealing at 55 °C for 30 s, and elongation at 72 °C for 30 s, and a final elongation at 72 °C for 5 min [25]. Amplicons from the same fermenter with different sampling days were pooled in equimolar. That is, 24 pooled amplicon samples were used in 2% agarose gel electrophoresis and then purified using an AxyPrep DNA Gel Extraction Kit (Axygen Biosciences, Union City, CA, USA). Amplicon libraries were generated using a NEB Next Ultra DNA Library Prep Kit for Illumina (New England Biolabs, Ipswich, MA, USA) according to the manufacturer′s recommendations, with the addition of index codes. Library quality was assessed on the Qubit 2.0 Fluorometer (Thermo Scientific) and Agilent Bioanalyzer 2100 system [37]. The library was sequenced on an Illumina MiSeq platform (2 × 250 bp) by Majorbio Company (Shanghai, China).

### 2.5. Sequencing Data Analysis

Raw fastq files were demultiplexed and quality-filtered using QIIME (Quantitative Insights into Microbial Ecology; version 1.19) with the following criteria: (1) the 250 bp reads were truncated at any site receiving an average quality score < 30 over a 10 bp sliding window, discarding the truncated reads that were shorter than 50 bp; (2) exact barcode matching, two nucleotide mismatch in primer matching, and reads containing ambiguous characters were removed; (3) only sequences that overlapped longer than 10 bp were assembled according to their overlap sequence [38]. In order to account for the differences in sequencing depth, all samples were randomly subsampled to 30,000 sequences prior to further analysis. Operational taxonomic units (OTUs) were clustered with 97% similarity cutoff using UPARSE (version 7.1; http://drive5.com/uparse/) and chimeric sequences were identified and removed using UCHIME. The taxonomy of representative sequences were analyzed using an RDP Classifier (http://rdp.cme.msu.edu/) against the Greengenes database (13_8) using a confidence threshold of 80% [39]. As two samples from one fermenter of the SHI and two samples from one fermenter of the CI had a large variation with other samples in the same group, they were excluded from further analysis. Thus, 20 samples from two sampling time points were used in the following sequence analysis. Good′s coverage, Shannon, Chao1, and the PD whole tree index were calculated for each sample using QIIME pipeline. The weighted UniFrac distance was calculated and used for principal coordinate analysis (PCoA). The significance of grouping in the PCoA plot was tested using analysis of similarity (ANOSIM) in QIIME with 999 permutations [40], and R varying between 0 and 1 described the strength of the treatments. Linear discriminant analysis (LDA) effect size (LEfSe) analysis was performed online (https://huttenhower.sph.harvard.edu/galaxy) to find significantly changed bacteria between groups using the criterion of LDA score higher than 3.0 [41].

### 2.6. Chemical Analysis

The concentration of NH_3_-N was determined using the phenol-hypochlorite procedure adapted from a previous study [42]. VFAs were determined using a GC system (Hewlett Packard Model 6890N, Agilent Technologies, Santa Clara, CA, USA) equipped with a DB-FFAP column (15 m × 0.25 mm × 0.25 µm) (Phenomenex, Florence, CA, USA) and a flame-ionization detector [43]. Helium served as carrier gas at a flow rate of 1.0 mL/min. The temperature was initially kept at 160 °C for 2 min and then increased to 220 °C at a rate of 5 °C/min; the temperature of the column was then maintained for 30 min. The temperature of both injector and detector was 280 °C. Isovalerate includes 2-methyl butyrate, which co-elutes. The GS activity was measured using a GS detection Kit (Jiancheng, Nanjing, China) according to the manufacturer’s instructions [44]. The methods of detecting GDH, GOGAT and ADH activities were the same as described by a previous study [44].

The contents of DM (method 930.15), OM (method 934.01) and CP (method 976.05) in the diet of rumen fluid donor-cows and filtered residues of effluent were determined according to the procedures of the Association of Official Analytical Chemists [45]. The NDF content was determined according to Van Soest et al. (1991) with a heat stable alpha-amylase and sodium sulfite and expressed inclusive of residual ash [46]. MPS in daily effluent was determined using the Kjeldahl method as described in AOAC (2000) [45]. Briefly, the effluent solution filtered with nylon cloth was centrifuged at 500× *g* for 10 min at 4 °C. The supernatant was centrifuged at 20,000× *g* for 20 min at 4 °C, and the pellet was washed with PBS buffer and centrifuged again. Then the microbial pellet was measured for nitrogen using a Kjeldahl analyzer (OPSIS LiquidLINE, Furulund, Sweden) to calculate the MPS. The efficiency of microbial protein synthesis (EMPS) was represented by grams of bacterial-N per kilogram of OM apparently digested in the fermenter [15]. Ruminal available N was calculated by subtracting undegraded N in effluent from N intake [47].

### 2.7. Statistical Analysis

Before analysis, the 16S rRNA gene copy numbers of total bacteria were transformed to log_10_ copy numbers to achieve normal distribution. Total bacteria number (represented by log_10_ 16S rRNA gene copy numbers), bacterial diversity indexes, fermentation parameters, VFAs, enzyme activities, MPS, EMPS, and the ratio of bacterial-N to available N in fermenters were statistically analyzed as repeated measures using the MIXED procedure of SAS 9.3 (SAS Institute, Cary, NC, USA). The two experimental periods were considered as two blocks. The model included treatment, days of sampling, their interaction, and block as fixed effects, and fermenter nested within (treatment × day of sampling) as a random effect [15]. Covariance structures, such as autoregressive (1), compound symmetry, unstructured, autoregressive, heterogeneous autoregressive, and heterogeneous compound symmetry, which had the lowest Akaike information criterion (AIC), were used in the covariance structures model [48]. Results were reported as least squares means. Means separation was conducted using Tukey′s test only when the main effect was significant. For pH and NH_3_-N data, mean separations were done for both daily and individual time points. The Spearman′s correlation among MPS, branched-chain volatile fatty acids (BCVFA), NH_3_-N (daily average values), enzyme activities (daily average values), and varied bacterial relative abundance (daily average values) were assessed using psych and corrplot packages in R (version 3.5.2). Correlations had an absolute Spearman’s correlation ≥ 0.50, with *p* < 0.05. The level of statistical significance was set at *P* < 0.05. A tendency for significance was declared at 0.05 ≤ *p* < 0.10.

### 2.8. Nucleotide Sequence Accession Number

All the raw sequences after assembling and filtering were submitted to the NCBI Sequence Read Archive (SRA; http://www.ncbi.nlm.nih.gov/Traces/sra/), under accession number SRP080973.

## 3. Results

### 3.1. In Vitro Bacterial Diversity

**Figure 2:**
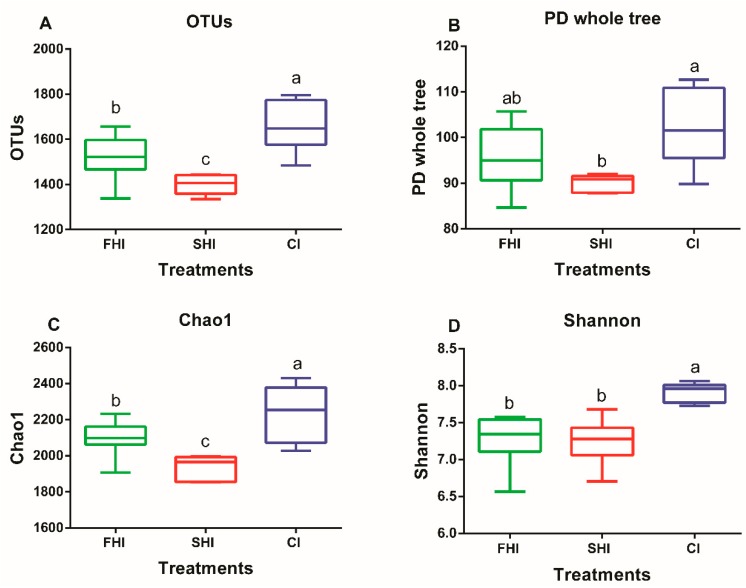
Alpha diversity index of rumen bacteria among treatments. Boxes represent the interquartile range (IQR) between the first and third quartiles (25th and 75th percentiles, respectively), and the horizontal line inside the box defines the median. Whiskers represent the lowest and highest values within 1.5 times the IQR from the first and third quartiles respectively. ^a–c^ Mean values with unlike letters were significantly different (*p* < 0.05). FHI, first half infusion of maltodextrin; SHI, second half infusion of maltodextrin; CI, continuous infusion of maltodextrin.

**Figure 3:**
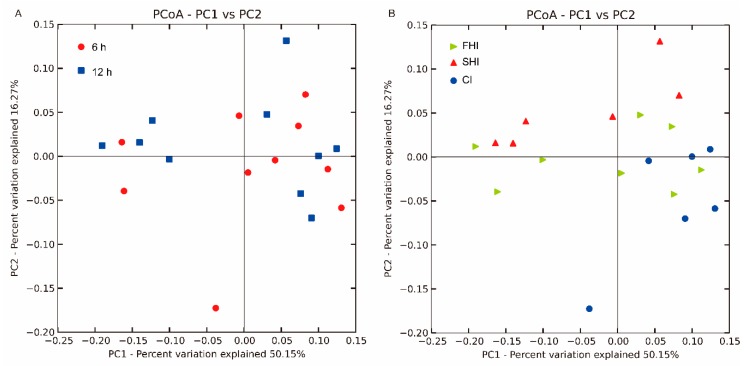
Principal coordinate analysis (PCoA) of the in vitro bacterial community. **A**, The comparison between sampling time. **B**, The comparison among treatments. 6 h, sampling at 6 h after feeding; 12 h, sampling at 12 h after feeding. FHI, first half infusion of maltodextrin; SHI, second half infusion of maltodextrin; CI, continuous infusion of maltodextrin.

A total of 2,527,740 high-quality sequences were obtained, and the remaining 2,488,817 sequences after chimeric removing were used to generate OTUs. In total, 3126 OTUs were kept in reference to the criterion of more than 5 sequences per OTU. As an indicator of microbial diversity, the Good′s coverage of all samples was more than 98%. The SHI had lower (*p* < 0.05) OTUs, PD whole tree, and Chao1 than the CI and FHI (Figure 2). The Shannon index was lower in the FHI and SHI (*p* < 0.01) than in the CI. As depicted in the Venn diagrams, the FHI and CI shared 312 OTUs, which were not included in the SHI (Figure S1). The distribution for the bacteria observed at two sampling times was similar (Figure 3A) as shown using the ANOSIM test (R = 0.052, *p* = 0.22). The ANOSIM test also showed that bacterial communities were different between the FHI and the SHI (R = 0.246, *p* = 0.02), and between the CI and the SHI (R = 0.517, *P* < 0.01), but similar between the FHI and the CI (R = 0.077, *p* = 0.20) (Figure 3B).

### 3.2. In Vitro Bacterial Composition

**Figure 4:**
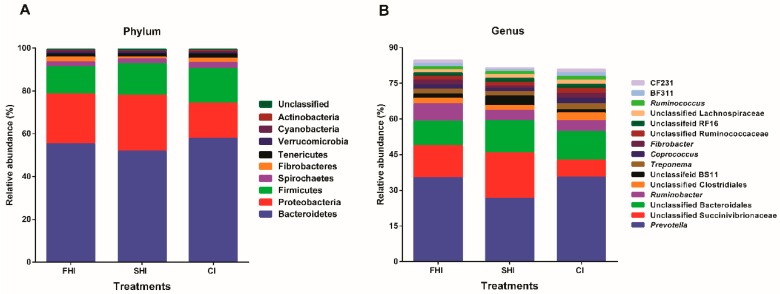
Effect of varying degree of synchronization on changes of microbial taxa. **A**, Relative abundance > 1% in phyla level. **B**, Top 15 (relative abundance) genera. FHI, first half infusion of maltodextrin; SHI, second half infusion of maltodextrin; CI, continuous infusion of maltodextrin.

**Figure 5:**
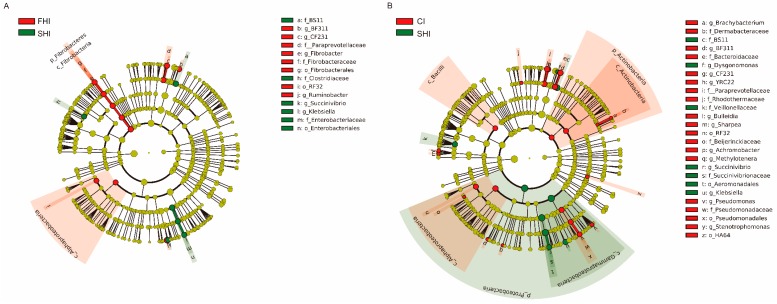
The differential phylogenetic distribution of bacterial communities between FHI and SHI (**A**) and between CI and SHI (**B**). LEfSe was used to determine the differentially relative abundance of bacterial taxa. Only LDA scores above 3 and P-value smaller than 0.05 were shown. LEfSe, linear discriminant analysis (LDA) effect size; FHI, first half infusion of maltodextrin; SHI, second half infusion of maltodextrin; CI, continuous infusion of maltodextrin.

*Bacteroidetes* (55.0% 16S rRNA gene reads), *Proteobacteria* (22.1%), and *Firmicutes* (14.3%) were the three predominant phyla (Figure 4A). *Prevotella* (32.4%), unclassified *Succinivibrionaceae* (13.2%), unclassified *Bacteroidales* (12.0%), *Ruminobacter* (5.4%), unclassified *Clostridiales* (2.6%), unclassified BS11 (2.3%), *Treponema* (2.2%), and *Fibrobacter* (1.7%) were the abundant genera (Figure 4B). LEfSe analysis showed that 17 and 32 taxa significantly changed between the FHI and the SHI, and between the CI and the SHI, respectively (Figure 5). The SHI had higher (LDA score ≥ 3.19) relative abundance of *Klebsiella* and *Succinivibrio*, and lower (LDA score ≥ 3.26) relative abundance of BF311 in *Bacteroidaceae*, CF231 in *Paraprevotellaceae*, *Fibrobacter*, and *Ruminobacter* than the FHI (Table 2). Compared with the CI, the SHI had higher (LDA score ≥ 3.01) relative abundance of *Dysgonomonas*, *Klebsiella*, and *Succinivibrio*, and lower (LDA score ≥ 3.02) relative abundance of BF311 in *Bacteroidaceae*, CF231 in *Paraprevotellaceae*, YRC22 in *Paraprevotellaceae*, *Pseudomonas*, and *Stenotrophomonas*.

### 3.3. In Vitro Fermentation and Nutrient Digestion

**Figure 6:**
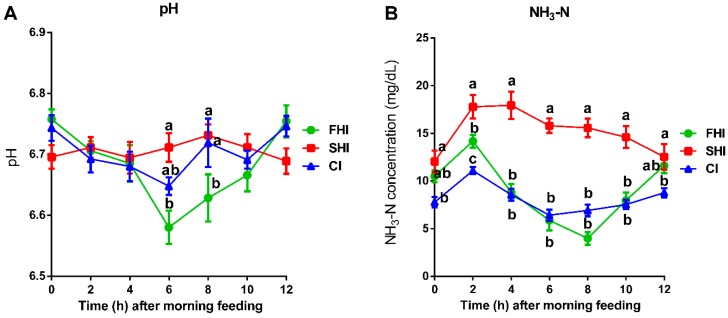
Effects of treatments with varying degrees of synchronization on fermenter pH and NH_3_-N concentration after morning feeding (09:00). Values are means, with standard errors represented by vertical bars. ^a–c^ Mean values with unlike letters were significantly different (*p* < 0.05). FHI, first half infusion of maltodextrin; SHI, second half infusion of maltodextrin; CI, continuous infusion of maltodextrin.

The average fermenter pH was similar (*p* = 0.62) among the three groups, while the average concentration of NH_3_-N in the SHI was greater (*p* = 0.01) than the FHI and CI (Table 3). The SHI group had greater (*p* ≤ 0.03) fermenter pH than the FHI at 6 and 8 h after feeding, and the SHI had greater (*p* ≤ 0.04) NH_3_-N than the FHI from 2 to 10 h after feeding (Figure 6). The FHI had similar NH_3_-N (*p* ≥ 0.86) from 4 to 12 h after feeding. The proportion of propionate was lower (*p* = 0.02) in the SHI and CI than that in the FHI at 2 h after feeding (Table 3). The proportions of BCVFA including isobutyrate and isovalerate were higher (*p* ≤ 0.01) in the SHI than that in the CI and FHI both at 2 and 10 h after feeding. However, there is no significant difference for other VFAs and digestibility of OM, DM, CP, and NDF among these three groups (Table S1).

### 3.4. Enzyme Activity and Microbial Protein Synthesis

**Figure 7:**
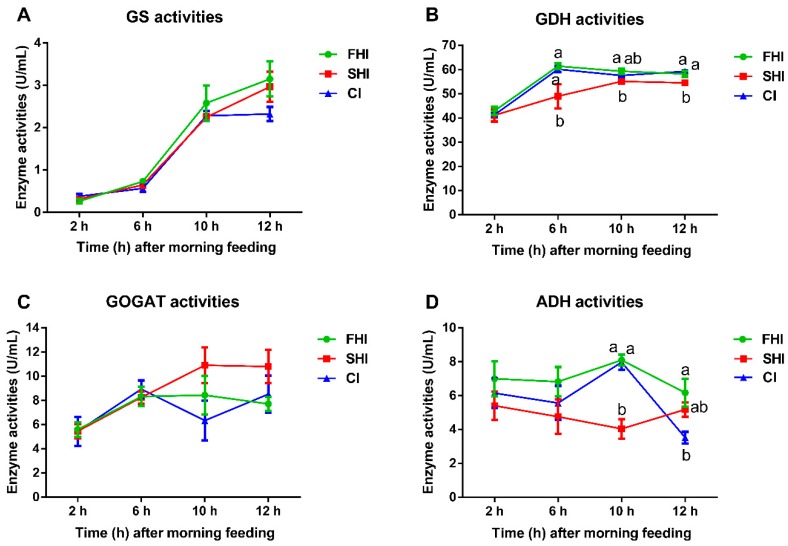
Activities of enzymes involved in ammonia assimilation among treatments. Values are means, with standard errors represented by vertical bars. ^a,b^ Mean values with unlike letters were significantly different (*p* < 0.05). GS, glutamine synthetase; GOGAT, glumate synthetase; GDH, glutamate dehydrogenase; ADH, alanine dehydrogenase. FHI, first half infusion of maltodextrin; SHI, second half infusion of maltodextrin; CI, continuous infusion of maltodextrin.

The GDH activity in the SHI was lower (*p* ≤ 0.04) than that in the FHI from 6 to 12 h after feeding, and the ADH activity in the SHI was lower (*p* < 0.01) than that in the FHI and CI only at 10 h after feeding (Figure 7). The FHI and CI had similar (*p* ≥ 0.67) GDH and ADH activities. The activities of GS and GOGAT were not affected (*p* ≥ 0.17) by different treatments. Compared with the FHI and CI, the SHI had lower (*p* ≤ 0.01) MPS, EMPS, and bacterial-N/available N (Table 4). Varying degrees of synchrony did not significantly affect total bacterial numbers at 6 and 12 h after feeding.

### 3.5. Correlations Between Bacterial Relative Abundance and Fermentation Parameters

**Figure 8:**
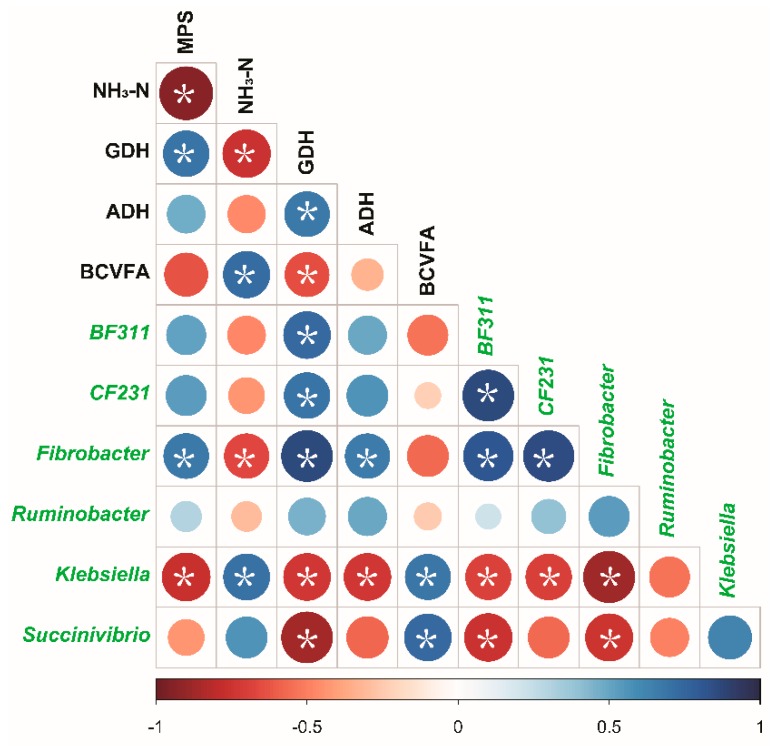
Relationship among MPS, NH_3_-N, enzyme activities, and bacterial relative abundance. Strong correlations are indicated by large circles, whereas weak correlations are indicated by small circles. Asterisks denote significant correlations (*p* < 0.05 and |r| ≥ 0.50). The colors of the scale bar denote the nature of the correlation with 1 indicating perfect positive correlation (dark blue) and -1 indicating the negative correlation (dark red). Microbes are marked by green. MPS, microbial protein synthesis; GDH, glutamate dehydrogenase; ADH, alanine dehydrogenase; BCVFA, branched-chain volatile fatty acids.

The correlative relationships among bacteria relative abundance, NH_3_-N, enzyme activities, and MPS were evaluated (Figure 8). The results showed that MPS was positively associated with GDH activity (r = 0.70, *p* = 0.03) and *Fibrobacter* (r = 0.67, *p* = 0.03) but was negatively associated with NH_3_-N (r = −0.94, *p* < 0.01) and *Klebsiella* (r = -0.77, *p* = 0.01). NH_3_-N was positively associated with BCVFA (r = 0.70, *p* = 0.02) and *Klebsiella* (r = 0.71, *p* = 0.02) and was negatively associated with GDH activity (r = -0.76, *p* = 0.01) and *Fibrobacter* (r = −0.66, *p* = 0.04). GDH activity was positively associated with BF211 (r = 0.73, *p* = 0.02), CF231 (r = 0.70, *p* = 0.03), and *Fibrobacter* (r = 0.87, *p* < 0.01) and was negatively associated with *Succinivibrio* (r = −0.87, *p* < 0.01) and *Klebsiella* (r = −0.73, *p* = 0.02). ADH activity was positively associated with *Fibrobacter* (r = 0.66, *p* = 0.04) and was negatively associated with *Klebsiella* (r = −0.73, *p* = 0.02).

## 4. Discussion

Synchronizing the rate of supply of ruminal energy and nitrogen sources was proposed as a potential method to improve MPS in ruminants [8,13]. According to previous studies [14,15,49], the release rate of energy other than protein mattered in synchrony. In situ rumen degradation showed that the water-soluble and rapidly degradable protein fraction of soybean meal is about 27%, and the in vitro crude protein degradation rate of soybean meal can be up to 60% in the first 6 h of incubation [50,51,52]. Okeke et al. (1983) also reported that the rumen NH_3_-N concentration was maximum at 0-6 h after feeding diets with soybean meal as the main protein source [53]. The same amount but different infusion rates and times of maltodextrin plus the carbohydrate in the basal diet were considered to mimic the different release rates of the energy matching fixed release rate of nitrogen (mainly from soybean meal) in a basal diet, and then to form varying degrees of synchronous diets in fermenters. The FHI, SHI, and CI were considered as a fast, slow, and continuous release of energy source in diets to form varying degrees of synchronization in this study, respectively. In order to avoid different amounts of energy source influencing fermentation, the same amount of maltodextrin was infused into the fermenters to achieve isoenergetic and isonitrogenous treatments per day. Thus, CI was fewer grams per hour than that for FHI or SHI. In addition, maltodextrin infusion was considered more relevant to practical farm diets because the main source of readily fermentable carbohydrate is usually starch, and maltodextrins would represent normal water-soluble intermediates in the ruminal degradation of starch [17,54,55]. As MPS was used as the standard to justify the degree of synchronization in our study, we expected that the SHI might have less MPS than the other two groups and was considered as the group having a lower degree of synchronization. In that case, the SHI was also expected to have a different microbial community than the FHI and CI.

In this study, *Bacteroidetes*, *Firmicutes*, and *Proteobacteria* were the most predominant bacteria and accounted for a total of 91.4% 16S rRNA gene reads in the fermenter. The genera, *Prevotella*, unclassified *Succinivibrionaceae*, *Ruminobacter*, *Treponema*, and *Fibrobacter* were the main bacteria. Among them, *Prevotella* and *Ruminobacter* are responsible for the utilization of starch [56,57]. *Fibrobacter* is known to be cellulolytic [58,59], and *Treponema* is a pectinolytic bacterium isolated from the bovine rumen [60]. As the amplicons from different sampling days were pooled together, the changed bacteria related to sampling day cannot be determined in this study.

The fermenter pH and NH_3_-N differed significantly across the different energy release rates, which was consistent with previous reports [12,61,62]. The difference of fermenter NH_3_-N concentrations showed that the in vitro N supplied by a basic diet and urea in the infusion buffer were utilized more rapidly in the FHI and CI than in the SHI. Therefore, the ability of in vitro microbial capture of N increased in the FHI and CI. A lower NH_3_-N concentration was also associated with more efficient utilization of N for MPS [63]. With more rapidly fermentable carbohydrate infused at 10 h after feeding, the propionate was higher in the FHI than that in the SHI. The increased BCVFA in the SHI might be due to the fact that more branched-chain amino acids were degraded as less energy and carbon skeleton were available in the diets at 2 and 10 h after feeding [64]. In accordance, Witt et al. (2016) also reported that VFA compositions were significantly affected by energy release rates [62]. We noticed that there was a daily effect for some items indicating that the fermenters might have some minor fluctuation during the sampling days. Thus, more adaptation and/or sampling days may be helpful to avoid these biases.

The ADH, GDH, GS, and GOGAT are key enzymes involved in the process of ammonia assimilation [65]. Even though the GS-GOGAT system has high affinity of ammonia, it is highly ATP-dependent and can be rapidly inactivated by an adenylation-deadenylation mechanism in an ample ammonia microenvironment [66,67]. In contrast, the GDH pathway plays a major assimilatory role with a comparatively low energy cost in an ample ammonia microenvironment [65]. ADH is extremely active during the process of ammonia assimilation with high concentrations of soluble carbohydrate and rumen ammonia [65]. In our study, the higher GDH and ADH activities reflected increased uptake of ammonia for MPS in the FHI and CI.

The SHI had lower MPS flow, EMPS, and bacterial-N/available N than the FHI and CI (*p* < 0.05). Previous works have also shown that carbohydrate infusion and a higher synchrony index can increase microbial growth, MPS and/or EMPS, and microbial N flow at the duodenum [12,17,61]. Furthermore, Henning et al. (1993) reported that a continuous infusion of energy resource resulted in a higher efficiency of microbial growth than that with pulse dosing [16]. MPS was closely associated with the number of microbes, protein content, and ammonia assimilation enzyme activities [65]. Even though the total bacterial numbers were similar in all treatments, GDH and ADH activities were higher in the FHI and CI. The lower MPS flow, EMPS, and bacterial-N/available N were consistent with the greater accumulation of NH_3_-N in the fermenter with the SHI than those in the fermenter with the FHI and CI, which may be explained by the lower NH_3_-N utilization efficiency and lower ADH and GDH activities in the fermenter. In accordance, the correlation also revealed the strong association among MPS, GDH, and NH_3_-N (Figure 8). A limitation of this study is that we did not measure the disappearance rate of maltodextrin in the fermenter, leading to open questions for future work, such as whether the fermentative parameters, especially pH, NH_3_-N, and MPS, will be altered when maltodextrin is infused faster in a short period of time. In addition, it will be helpful to build a better model of the energy and nitrogen release in the fermenter by using the disappearance rate of maltodextrin. Therefore, the amounts of energy and protein sources, as well as the release rates and matching degrees of these sources, need to be equally considered in practical diet formulating.

The varied bacteria identified by LEfSe and their correlations with MPS were highly consistent, where the biomarkers of the FHI and CI were all positively associated with MPS, while the biomarkers of the SHI were all negatively associated with MPS. It was reported that NH_3_-N is the sole nitrogen source of *Fibrobacter* [68]. Matheron et al. (1999) found that GDH and ADH were the pathways of ammonia assimilation in *Fibrobacter*, but GS activity was very low or even undetectable in *Fibrobacter* [69]. As a starch degrader, *Ruminobacter* was largely increased in FHI and CI, which had higher MPS [57]. Indeed, NH_3_-N is also the essential nitrogen source of *Ruminobacter* in the rumen other than amino acids or peptides [57,70]. As no GOGAT activity was detected in *Ruminobacter*, the GS-GOGAT couple could not function, and as a consequence, GDH became the main ammonia assimilation enzyme which was accompanied by an active accumulation of ammonia intracellularly [70]. Therefore, the fermenter NH_3_-N concentration, GDH activity, and these bacteria were the main indicators of MPS. The genera BF311 and CF231 were also identified as biomarkers in the FHI and CI and were positively associated with *Fibrobacter*. Although the function of them has been little studied, they were found in many rumen samples and some studies showed their relative abundance might change with forage-based diets [54,71,72], hinting a potentially crucial role in the rumen ecosystem even in ruminal synchrony.

Even though the GS-GOGAT system for ammonia assimilation was found in *Klebsiella*, that pathway is highly ATP-dependent and energy consuming as two ATP were needed for the cyclic NH_3_/NH_4_^+^ retention of each molecule NH_3_ [70,73]. For this reason, until now the GS-GOGAT system has only been found to be expressed in *Klebsiella* aerogenes [74]. Even though GDH activity was also found in *Klebsiella*, some mutants of *Klebsiella aerogenes* lack GDH activity [74,75]. As the relative abundance of *Klebsiella* was greater in less synchronous fermenters (SHI) than in more synchronous fermenters (FHI and CI) in this study, it can be reasonably inferred that inefficient transformation from GS to GDH activity in *Klebsiella*, or less (even lack of) GDH activity, resulted in higher NH_3_-N in less synchronous fermenters and then was responsible for the lower MPS and EMPS. Belonging to the same family as *Ruminobacter*, *Succinivibrio* was also reported to be an amylolytic bacteria in rumen [57]. The decreased relative abundance of *Succinivibrio* in FHI and CI might be due to its lower competitive ability compared with starch-degrading bacteria, such as *Ruminobacter* [57]. *Ruminobacter* also has the ability to degrade urea and protein and use amino acids as a nitrogen source in rumen [70,76]. Hence, it is reasonable to infer that *Ruminobacter* might be responsible for the degradation of branched-chain amino acids into BCVFA in our study. Even though some bacteria such as *Fibrobacter*, *Ruminobacter*, *Succinivibrio*, and *Klebsiella* have a lower relative abundance and/or biomass yield compared to most other rumen bacteria, they may associate with other microbe and exert more influence on the rumen community structure and ecosystem functioning [19,77,78]. It should be noted that some correlations do not guarantee causality and more work is needed to illustrate the integrated functional characteristics of these microbial communities.

## 5. Conclusions

**Figure 9:**
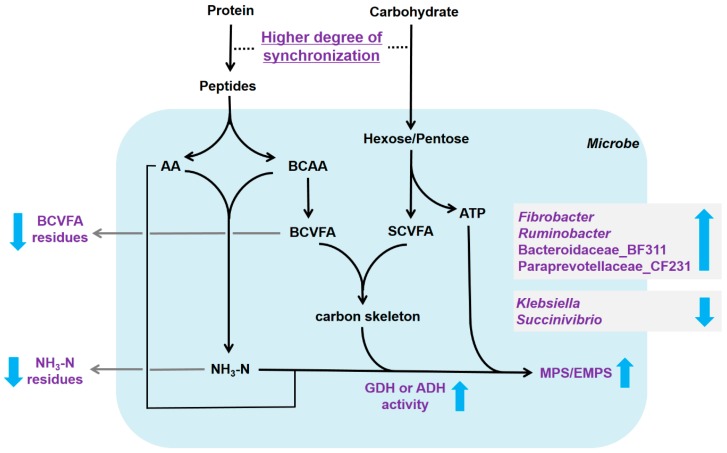
Diagram of how synchrony in the release of energy and protein influences the microbial synthesis pathways. The Cambridge blue area indicates the intracellular pathways of rumen bacteria. The light purples components indicate significant changes in our study, and the sideward arrows mean increased or decreased. AA, amino acids; ADH, alanine dehydrogenase; ATP, adenosine triphosphate; BCAA, branched-chain amino acids; BCVFA, branched-chain volatile fatty acids; GDH, glutamate dehydrogenase; MPS, microbial protein synthesis; EMPS, efficiency of microbial protein synthesis; SCFA, short-chain fatty acids.

Taken together, a higher degree of synchronization of energy and nitrogen release leads to active ammonia assimilation and higher MPS and EMPS, partially by increasing the energy supply and GDH and ADH activity in vitro, which results in lower NH_3_-N and BCVFA residues (Figure 9). During this metabolism, the relative abundance of *Fibrobacter* and *Ruminobacter* increases, while that of *Klebsiella* and *Succinivibrio* decreases. Therefore, the changing degrees of synchronization of energy release rate and bacterial community may be a potential manipulation point in practice to increase MPS and EMPS, and then to improve ruminant productivity. In addition, the amounts, release rates, and matching degrees of energy and protein sources need to be considered in practical diet formulating.

## Figures and Tables

**Table 1:** Ingredient and nutrient composition of total mixed ration for rumen fluid donated cows and of basic diet for rumen simulation system (RSS).

Items ^1^, % DM	Cow Diet ^3^	RSS Diet
Corn silage	22.4	
Alfalfa hay	14.5	
Chinese wildrye	8.8	
Corn stover		46.25
Ground corn	22.9	25.00
Soybean meal	9.7	28.09
Cottonseed meal	4.6	
DDGS	4.6	
Whole cottonseed	8.9	
Fat powder	0.4	
Salt	0.5	
Premix ^2^	2.5	0.66
Chemical composition ^1^, % DM		
CP	15.5	19.2
RDP	8.4	12.3
NDF	30.2	43.7
NFC	39.0	29.5
Starch	26.1	20.8

^1^ DDGS, distiller dried grains with solubles; DM, dry matter; CP, crude protein; RDP, rumen degradable protein, NDF, neutral detergent fiber; NFC, nonfibrous carbohydrate = 100 – (NDF + CP + ether extract + ash). ^2^ Premix contained (DM basis) 99.07% of ash, 14.27% of Ca, 5.42% of P, 4.96% of Mg, 0.05% of K, 10.67% of Na, 2.98% of Cl, 0.37% of S, 11 mg/kg of Co, 577 mg/kg of Cu, 4,858 mg/kg of Fe, 51 mg/kg of I, 1806 mg/kg of Mn, 13 mg/kg of Se, 1694 mg/kg of Zn, 115,240 IU/ kg of vitamin A, 46,100 IU/kg of vitamin D, and 576 IU/kg of vitamin E. ^3^ Total mixed ration of rumen fluid donated cows.

**Table 2:** The relative abundance (%) of significantly changed taxa between FHI and SHI and between CI and SHI.

Comparison	Items	Taxa (Phylum; Family; Genus)	Relative Abundance in Treatments, %			LDA Score (log_10_)
			FHI	SHI	CI	
FHI vs. SHI	FHI enriched	Bacteroidetes; Bacteroidaceae; BF311	1.39	0.78	1.56	3.50
		Bacteroidetes; Paraprevotellaceae; CF231	1.32	0.97	1.35	3.26
		Fibrobacteres; Fibrobacteraceae; *Fibrobacter*	2.10	0.97	2.01	3.77
		Proteobacteria; Succinivibrionaceae; *Ruminobacter*	7.19	4.25	4.61	4.18
	SHI enriched	Proteobacteria; Enterobacteriaceae; *Klebsiella*	0.28	1.24	0.44	3.73
		Proteobacteria; Succinivibrionaceae; *Succinivibrio*	0.20	0.49	0.23	3.19
CI vs. SHI	CI enriched	Bacteroidetes; Bacteroidaceae; BF311	1.39	0.78	1.56	3.58
		Bacteroidetes; Paraprevotellaceae; CF231	1.32	0.97	1.35	3.25
		Bacteroidetes; Paraprevotellaceae; YRC22	0.71	0.64	0.88	3.08
		Proteobacteria; Pseudomonadaceae; *Pseudomonas*	0.17	0.09	0.32	3.09
		Proteobacteria; Xanthomonadaceae; *Stenotrophomonas*	0.02	0.01	0.02	3.02
	SHI enriched	Bacteroidetes; Porphyromonadaceae; *Dysgonomonas*	< 0.01	0.03	0.01	3.01
		Proteobacteria; Enterobacteriaceae; *Klebsiella*	0.28	1.24	0.44	3.61
		Proteobacteria; Succinivibrionaceae; *Succinivibrio*	0.20	0.49	0.23	3.13

FHI, first half infusion of maltodextrin; SHI, second half infusion of maltodextrin; CI, continuous infusion of maltodextrin; LDA, linear discriminant analysis.

**Table 3:** Effect of treatments with varying degrees of synchronization on in vitro fermentation parameters.

Time	Items	Treatments			SEM	*p*-Value		
		FHI	SHI	CI		Treatment	Day	Treatment × Day
Daily	pH ^1^	6.68	6.71	6.70	0.027	0.62	<0.01	0.53
	NH_3_-N, mg/dL ^1^	9.17 ^b^	15.28 ^a^	8.16 ^b^	2.126	0.01	<0.01	0.01
2 h	TVFAs, mmol/L	77.58	72.31	79.51	2.489	0.05	0.98	0.44
	Acetate, %	62.92	62.27	63.57	1.104	0.72	0.65	0.57
	Propionate, %	20.91	19.95	20.08	0.860	0.66	0.07	0.31
	Butyrate, %	11.25	11.85	11.82	0.432	0.52	0.85	0.78
	Valerate, %	2.15	2.15	2.15	0.118	0.87	0.03	0.26
	Isobutyrate, %	0.70 ^b^	0.84 ^a^	0.60 ^a^	0.035	0.01	0.04	0.34
	Isovalerate, %	2.06 ^b^	2.88 ^a^	1.81 ^b^	0.178	0.01	0.01	0.06
	BCVFA, %	2.76 ^b^	3.72 ^a^	2.41 ^b^	0.187	<0.01	<0.01	0.06
10 h	TVFAs, mmol/L	56.26	60.85	58.93	3.892	0.69	0.55	0.25
	Acetate, %	62.87	63.33	64.19	1.529	0.81	0.08	0.30
	Propionate, %	20.83 ^a^	17.16 ^b^	18.22 ^b^	0.915	0.02	0.01	0.01
	Butyrate, %	11.89	12.41	12.68	0.757	0.73	0.08	0.75
	Valerate, %	2.00	2.03	2.21	0.127	0.44	0.97	0.65
	Isobutyrate, %	0.619 ^b^	0.76 ^a^	0.62 ^b^	0.033	0.01	0.82	0.28
	Isovalerate, %	1.78 ^b^	4.32 ^a^	2.08 ^b^	0.174	<0.01	0.01	0.10
	BCVFA, %	2.40 ^b^	5.07 ^a^	2.71 ^b^	0.181	<0.01	0.01	0.11

^a,b^ Mean values with unlike letters were significantly different (*p* < 0.05). ^1^ Average value per day. TVFAs, total volatile fatty acids; BCVFA, branched-chain volatile fatty acids; OM, dry matter; CP, crude protein; NDF, neutral detergent fiber; SEM, standard error of means; FHI, first half infusion of maltodextrin; SHI, second half infusion of maltodextrin; CI, continuous infusion of maltodextrin.

**Table 4:** Effect of degrees of synchronization on microbial protein synthesis and total bacterial counts.

Items	Treatments			SEM	*p*-Value		
	FHI	SHI	CI		Treatment	Day	Treatment × Day
MPS, g CP/d	6.29 ^a^	4.45 ^b^	6.55 ^a^	0.219	<0.01	<0.01	0.19
EMPS ^1^	40.47 ^a^	29.23 ^b^	40.29 ^a^	1.321	0.01	0.13	0.31
Bacterial-N/available N ^2^, %	49.02 ^a^	34.74 ^b^	49.19 ^a^	1.612	<0.01	0.07	0.31
Total bacteria, log_10_ 16S rRNA gene copy numbers/mL fermenter fluid							
6 h	10.92	10.90	11.26	0.297	0.59	0.64	0.99
12 h	11.97	11.38	11.98	0.220	0.10	0.64	0.18

^a,b^ Mean values with unlike letters were significantly different (*p* < 0.05). ^1^ CP, crude protein; EMPS = efficiency of microbial protein synthesis (grams of bacterial-N per kilogram of OM apparently digested in the fermenter) [15]. ^2^ Available N = N intake—undegraded N [47]. MPS, microbial protein synthesis; SEM, standard error of means; FHI, first half infusion of maltodextrin; SHI, second half infusion of maltodextrin; CI, continuous infusion of maltodextrin.

## Supplementary Materials

The following are available online at https://www.mdpi.com/2076-2607/8/2/231/s1. Table S1: Effect of treatments with varying degrees of synchrony on in vitro apparent nutrient digestion; Figure S1: Venn map for operational taxonomic units (OTUs) identified from treatments.
