# Supplementary material for: Synchrony Degree of Dietary Energy and Nitrogen Release Influences Microbial Community, Fermentation, and Protein Synthesis in a Rumen Simulation System
Source: Microorganisms. 2020 Feb 9;8(2):231. doi: 10.3390/microorganisms8020231 (PMC7074744; doi:10.3390/microorganisms8020231)
Supplement: Supplementary file 1 [file microorganisms-08-00231-s001.zip › Supplementary Table S1 20191126 Effect of treatments with varying degrees of synchronization on in vitro digestibility.docx]

**Supplementary files:**

**Table S1.** Effect of treatments with varying degrees of synchrony on *in vitro* apparent nutrient digestion

| Items | Treatments | | | SEM | *P*-value | | |
| --- | --- | --- | --- | --- | --- | --- | --- |
|  | FHI | SHI | CI |  | Treatment | Day | Treatment × day |
| OM, % | 62.2 | 61.2 | 65.0 | 3.16 | 0.49 | < 0.01 | 0.04 |
| CP, % | 68.2 | 68.2 | 71.5 | 3.62 | 0.60 | < 0.01 | < 0.01 |
| NDF, % | 25.0 | 29.9 | 26.6 | 1.96 | 0.18 | 0.33 | 0.37 |

OM, dry matter; CP, crude protein; NDF, neutral detergent fiber; SEM, standard error of means; FHI, first half infusion of maltodextrin; SHI, second half infusion of maltodextrin; CI, continuous infusion of maltodextrin.
