# Supplementary figures and images for: Synchrony Degree of Dietary Energy and Nitrogen Release Influences Microbial Community, Fermentation, and Protein Synthesis in a Rumen Simulation System
Source: Microorganisms. 2020 Feb 9;8(2):231. doi: 10.3390/microorganisms8020231 (PMC7074744; doi:10.3390/microorganisms8020231)

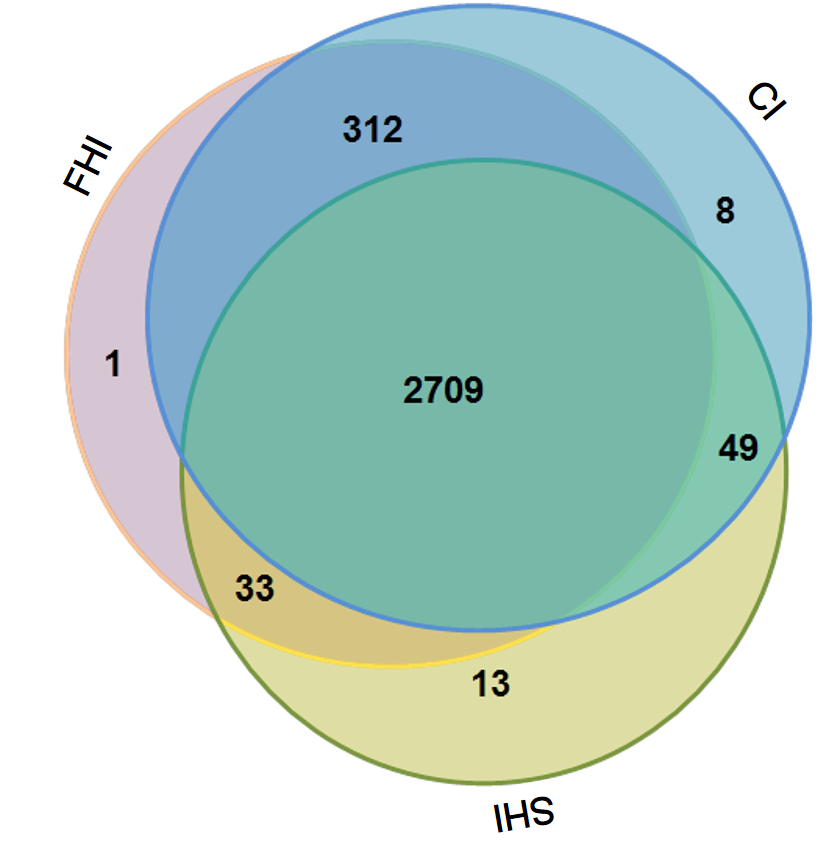

Supplement: Supplementary file 1 [file microorganisms-08-00231-s001.zip › FS1 vennmap.tif]
